# Editorial: Citrus viruses: pathogenicity, genetic variation and molecular biology

**DOI:** 10.3389/fpls.2023.1325570

**Published:** 2024-01-04

**Authors:** Yasir Iftikhar, Abdullah M. Al-Sadi, Sagheer Atta

**Affiliations:** ^1^ Department of Plant Pathology, College of Agriculture, University of Sargodha, Sargodha, Pakistan; ^2^ Department of Crop Sciences, College of Agricultural and Marine Sciences, Sultan Qaboos University, Muscat, Oman; ^3^ Department of Plant Protection, Faculty of Agricultural Sciences, Ghazi University, Dera Ghazi Khan, Pakistan

**Keywords:** citrus viruses, tristeza, citrus aphid, detection, psyllid, Huanglongbing

Citrus is one of the most economically important fruit tree crops in the tropical and subtropical areas of the world. Citrus fruits have high nutritional and medicinal values including the vitamins and antioxidants. China is the top citrus producing country. Like other crops, many diseases caused by different pathogens are the nightmare for citrus production. Among them the virus and virus-like pathogens are the major constraints in citrus production. Most of the citrus viruses and virus-like pathogens are graft-transmissible and can be transmitted via insect vectors. Citrus virology has been a neglected area in the recent past. *Citrus tristeza virus* (CTV) and Citrus greening disease (CGD) are the major threat for citrus production in terms of yield and quality. Both CTV and CGD have been a limiting factor for the citrus industry in tropical and subtropical areas of the world. These diseases have been a potent cause of not only citrus trees debilitation but also the vanishing of citrus orchards. There are different research groups working on citrus viruses in different parts of the world. The research areas are diverse, ranging from identification of citrus viruses to the epidemiology and management of viral diseases. Research and recent advances in these areas provided a solid background for the long-term management of plant viruses. Keeping in view the importance of citrus viruses the topic entitled, “*Citrus viruses: Pathogenicity, Genetic variation and Molecular biology*” was selected for the Research Topic with the aim to provide a platform to plant scientists working on the pathogenicity, biology, transmission and genetic variation of citrus viruses and virus-like pathogens. This topic includes six articles, with more focus on CTV.

Liu et al. shed the light on new methods of identification and classification of citrus diseases which are different from conventional methods. The article focused on an image enhancement method based on the MSRCR algorithm and homomorphic filtering algorithm optimized by Laplacian (HFLF-MS) to highlight the disease characteristics of citrus. Moreover, it highlighted a new neural network DS-MENet based on the DenseNet-121 backbone structure. The combination of these methods provided a strong base for the classification of citrus diseases. Similarly, Khalilzadeh et al. emphasized the significance of the complex nature of virus-associated stem pitting in perennial woody plants. They described the comparative transcriptome of different stem pitting isolates of CTV infecting citrus. Their study showed a little variation in the expressed genes (DEGs) in mild isolated as compared to the sever isolated of CTV, which showed substantial gene expression changes. The findings of this study, thus, provide evidence for the association between stem pitting phenotypes and SVT regeneration, suggesting that the expression of these genes might play important roles in the development of stem pitting symptoms.

Shen et al. article focused on Huanglongbing (HLB), one of the most economically devastating diseases in citrus industry, by investigating their polypeptides associated with pathogenicity. They proposed that a *Candidatus* Liberibacter asiaticus (CLas)-secreted Sec-dependent polypeptide, namely SECP8 (CLIBASIA_05330), may serve as a novel broad-spectrum suppressor of plant immunity, and provide the first evidence counteractive effect among CLas effectors.

Biswas et al. reported the use of a mild strain of CTV for cross protection in Grapefruit trees in South Africa. They used grapefruit mild strain 12 (GFMS12) as the source for single aphid transmissions (SAT) using brown citrus aphid (BrCA). The transmitted CTV sub-isolates were analyzed via serological and molecular assays. The RT-PCR reported nine CTV genotypes in the complex of severe and mild genotypes including GFMS12. Among them a single BrCA transmitted up to six CTV genotypes simultaneously. They also concluded that the heteroduplex mobility assay has been found to be a rapid, reliable tool for the identification of genotypes. Britt-Ugartemendia et al. emphasized the destructive nature of citrus tristeza disease and Huanglongbing in Florida. They found the CTV in citrus samples and Asian citrus psyllid (*Diaphorina c*itri) (ACP) during a survey in citrus orachrds of Florida. They concluded that presence of CTV in ACP could be a tool to identify the CTV in citrus orchards. Qin et al. reported that Citrus chlorotic dwarf-associated virus (CCDaV) is a major a threat to the citrus industry in China. They investigated that the CCDaV-RepA elicits a hypersensitive response (HR)-like cell death in an indicator host, *Nicotiana benthamiana*. They also reported the production of H2O2 and ion leakage. They concluded the interaction of host and CCDaV-RepA proteins during the infection process of CCDaV in citrus.

## Author contributions

YI: Writing – original draft. AA-S: Writing – review & editing. SA: Writing – review & editing.

